# Topology-directed silicide formation: an explanation for the growth of C49-TiSi_2_ on the Si(100) surface

**DOI:** 10.1039/d6na00467a

**Published:** 2026-08-07

**Authors:** Lukas Hückmann, Jonathon Cottom, Jörg Meyer, Emilia Olsson

**Affiliations:** a Advanced Research Center for Nanolithography Science Park 106 1098 XG Amsterdam The Netherlands e.olsson@arcnl.nl; b Institute for Theoretical Physics, University of Amsterdam Science Park 904 1098 XH Amsterdam The Netherlands; c Leiden Institute of Chemistry, Gorlaeus Laboratories, Leiden University P.O. Box 9502 2300 RA Leiden The Netherlands

## Abstract

Designing metal–semiconductor (MS) junctions is essential for optimizing the performance of modern nanoelectronic devices. A widely used material is TiSi_2_, which combines low electronic resistivity with good endurance. However, its multitude of polymorphs poses a challenge for device fabrication. Although the low-resistivity C54-TiSi_2_ is thermodynamically favored, silicide formation on Si(100) nucleates through the metastable C49-TiSi_2_ phase, whose unfavorable electronic properties limit device performance. The origin of this phase selection remains poorly understood despite the ubiquity of TiSi_2_ in nanoelectronic devices. Based on extensive density functional theory calculations, we present a comprehensive model of Ti adsorption on Si(100) that highlights the pivotal role of surface topology for the initial stages of the interfacial TiSi_2_ formation process. We show that the interplay between Si surface dimers, the symmetry of the Si(100) surface, and the incorporation of Ti adsorbates below the surface drives an adsorption pattern that yields a nucleation template for the C49-TiSi_2_ phase. This novel perspective on the unique growth of TiSi_2_ will not only pave the way for next-generation electronic devices, but also demonstrates how silicon's surface topology can be a decisive factor for optimizing MS-junctions at the nanoscale.

## Abstract 2

TiSi is widely used in metal–semiconductor junctions, but formation of the unfavorable C49 polymorph limits its use. DFT reveals that Si(100) surface topology acts as a nucleation template, enabling a unique growth mechanism for the undesired phase.

## Graphical abstract

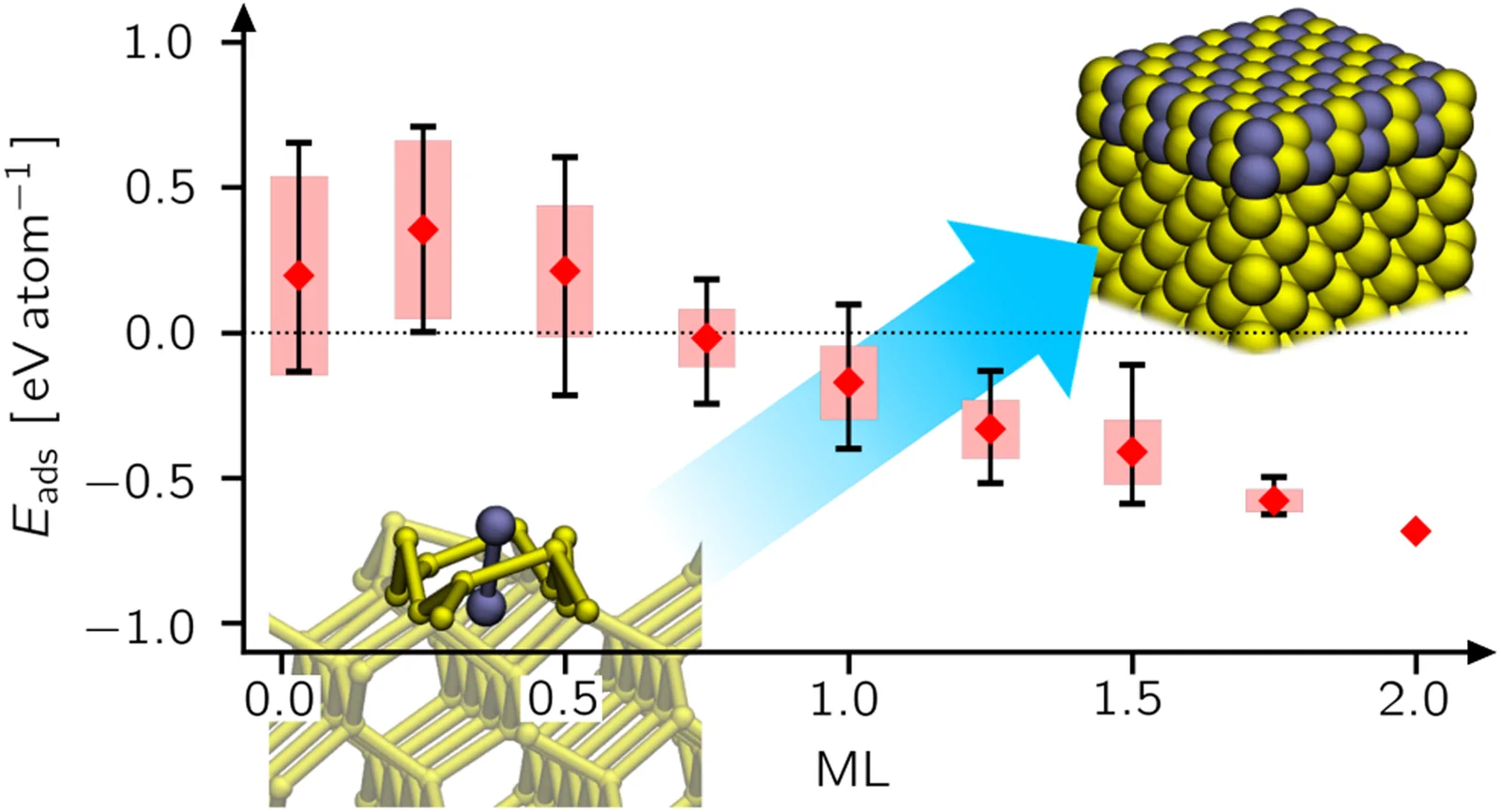

## 1 Introduction

The performance of modern micro- and optoelectronic devices hinges on reliable metal–semiconductor contacts at nanometer scales. Among available contact materials, titanium disilicide (TiSi_2_) is widely used in Si-based architectures due to its low electrical resistivity, small Schottky barrier on n-type Si, and chemical stability.^1–4^ These properties have established TiSi_2_ as an important material for CMOS technologies, Schottky diodes, and photodetectors,^5–9^ and have prompted exploration of its role in emerging applications, including photocatalytic water splitting^10–13^ and perovskite-Si tandem photovoltaics.^14,15^

Yet, despite its technological ubiquity, the fabrication of phase-pure TiSi_2_ remains a persistent challenge. Upon annealing, Ti deposited on crystalline Si forms a metastable orthorhombic C49 phase, which only transforms to the desired low-resistivity C54 polymorph at elevated temperatures.^16–19^ The persistent nucleation of C49-TiSi_2_ – even under conditions where C54 is thermodynamically favored^20^ – suggests a complex and poorly understood interplay of interfacial energetics, surface mobility, and local strain. The limitations of C49 not only degrade the electrical performance through increased contact resistance, but also impose stringent thermal budgets that constrain integration with temperature-sensitive materials in advanced device stacks,^9,19,21^ such as 3D architectures and backside power delivery networks.^22–24^ Understanding and ultimately controlling this phase selection is thus critical for enabling next-generation nanoscale logic^25–28^ and optoelectronic platforms.^12,14,29–31^

In standard device processing, thin TiSi_2_ layers are formed *via* self-aligned silicidation (salicidation), where Ti is deposited onto single-crystalline Si and subsequently annealed.^32^ Initial Ti growth follows a Stranski–Krastanov-like mode:^33,34^ after formation of a monolayer, additional adatoms cluster into three-dimensional islands. Notably, XPS measurements reveal the onset of silicide formation even at room temperature,^35,36^ suggesting immediate interfacial reactivity upon deposition. Upon thermal activation, Ti diffuses into the Si substrate and reacts to form silicide phases. The binary Si–Ti system exhibits a rich phase diagram with multiple low-symmetry compounds and stoichiometries,^37,38^ with the resulting structure being dictated by both temperature and annealing duration. At approximately 600 °C, the orthorhombic C49-TiSi_2_ polymorph (space group *Cmcm*) nucleates, which transforms into the stable C54 phase (*Fddd*) above 700 °C.^39^ The C54 polymorph is technologically important due to its lower resistivity (15–20 µΩ cm *vs.* 60–80 µΩ cm for C49)^17,40^ and higher thermodynamic stability compared to C49 (ΔΔ*H*_f_ ≈ 2–4 kJ mol^−1^).^18,20^

Despite these insights, salicidation invariably produces C49-TiSi_2_ as the initial phase,^16^ necessitating complex, energy-intensive multi-step annealing procedures to induce C54 formation and heal microstructural defects that would otherwise compromise yield and long-term reliability. The unresolved origin of the detrimental formation sequence of these two phases represents a significant bottleneck in fully harnessing TiSi_2_'s potential for scaled technologies. Various studies have attributed the preference for C49 to its lower surface energy^41,42^ or enhanced Ti diffusivity within its lattice,^43^ indicating a kinetically driven process. Yet, these hypotheses remain inconclusive, and a consistent atomic-scale explanation for first forming the C49 phase is lacking.^2,44^

To address this gap, several atomistic simulation studies have examined TiSi_2_ formation at the Si interface. Early work by Yu *et al.*^45^ modeled Ti deposition on an unreconstructed 1 × 1 Si(100) surface and reported that interfacial energetics favor C49-TiSi_2_ over C54. Subsequent studies investigated Ti adsorption and adatom diffusion,^46–48^ showing that the Si(100) surface reconstruction with the surface atoms forming dimers strongly influences adatom clustering and adsorption energetics, though high surface coverages and silicide intermixing were not investigated. More recent efforts have focused on the bulk and defect thermodynamics: Sánchez *et al.*^49^ assessed the formation energies of Ti defects in crystalline Si, while Brown *et al.*^44^ systematically evaluated the phase stability of the C49 and C54 polymorphs. Collectively, these studies underscore the delicate energetic balance at the Si–Ti interface, but an understanding of how atomic-scale processes drive C49 nucleation remains elusive. This phenomenon exemplifies a broader class of interface-driven metastability in thin-film heterostructures, where transient polymorphs emerge and persist despite being thermodynamically disfavored – a recurring theme across material systems such as silicides, nitrides, and transition-metal chalcogenides.^50–54^

In this paper, we perform density functional theory calculations to develop a comprehensive model for the initial stages of Ti growth on Si(100). Starting from isolated Ti adsorbates and extending to coverages of two monolayers, we systematically identify preferred adsorption sites, interaction mechanisms, and the conditions for which adsorbed Ti–Ti pairs lift the *c*(4 × 2) Si(100) surface reconstruction. We show that these local structural motifs disrupt Si dimers and drive the self-promoting formation of a TiSi bilayer. Remarkably, this bilayer reproduces the low-symmetry zigzag motifs unique to the C49 polymorph, thereby acting as a nucleation template for its growth. Our findings reveal that the emergence of C49 is governed primarily by surface topology rather than by enhanced diffusion or interfacial stress as previously assumed. This provides a unified framework for understanding Stranski–Krastanov growth and paves the way towards suppression of the C49 phase by surface disruption, preamorphization, or refractory metal overlayers.

## 2 Results and discussion

### 2.1 Ti-deposition

**Fig. 1:**
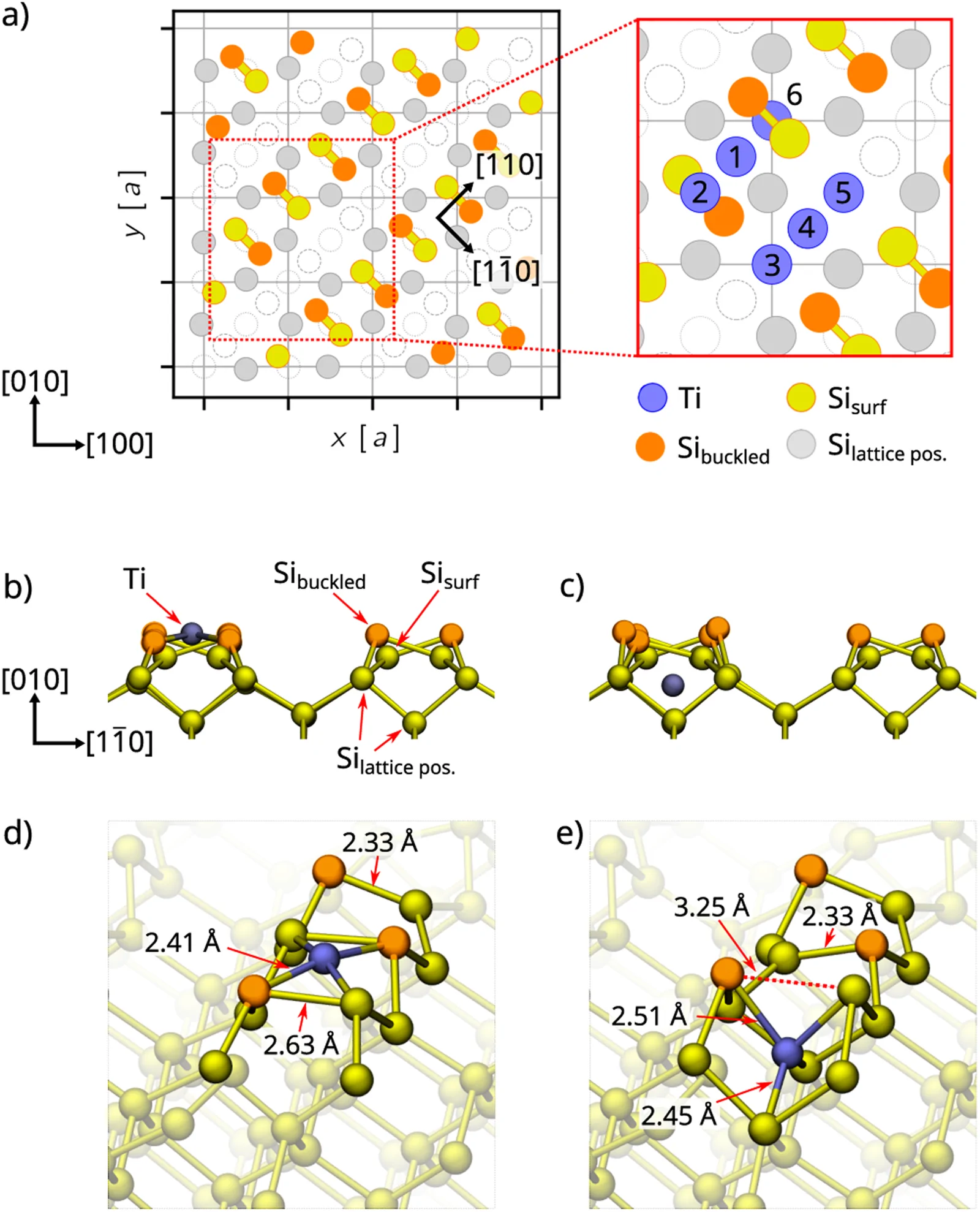
(a) Schematic of the Ti adsorption sites on the *c*(4 × 2) Si(100) surface (top view). The numbered blue circles in the inset represent Ti adatoms, the orange and yellow ones represent buckled Si surface dimers with the orange ones being elevated, and the gray (hollow) ones are the Si atoms of subsurface layers on their lattice positions, respectively. (b) and (c) Side view of the Ti adsorption sites at position 1 and 6. (d) and (e) Depiction of a Ti atom adsorbed at position 1 and 6 with bond lengths showcasing the local reversal of the Si surface reconstruction. Si is colored yellow, Ti blue.

The pristine Si(100) surface consists of two surface silicon atoms per surface unit cell, each with two-fold coordination. To maximize coordination, these surface atoms reconstruct by tilting and forming dimers with neighboring atoms. The resulting Si dimers are not perfectly parallel to the surface but exhibit an asymmetric buckling, giving rise to various possible surface symmetries. Comparing the energetics of different surface configurations confirms that the *p*(2 × 2) and *c*(4 × 2) reconstructions represent the lowest-energy configurations, consistent with STM measurements^55,56^ and earlier computational studies.^57,58^ The Si dimers align parallel to each other along the [110]-direction on the surface forming characteristic patterns of Si-dimer ridges and trenches (see Fig. 1a, yellow/orange sites *vs.* hollow circles). Further details on the surface reconstructions are provided in the SI, Section 1.

On the *c*(4 × 2) Si(100) surface, five high-symmetry adsorption sites can be identified, as illustrated in Fig. 1a: the four-fold coordinated hollow site between two dimers (1), on a bridge site centered on a buckled dimer (2), and three trench sites between dimers (3–5). The Ti adsorption energies (*E*_ads_) are referenced to bulk α-Ti, so that *E*_ads_ < 0 indicates that Ti is energetically more stable in the Ti–Si configuration than in elemental Ti. Among these, only Ti^(^^1^^)^ and Ti^(^^6^^)^ are favorable compared to α-Ti, with *E*_ads_(Ti^(^^1^^)^) = −0.06 eV and *E*_ads_(Ti^(^^6^^)^) = −0.14 eV, while all the others are significantly higher in energy. Ti^(^^4^^)^ is unstable and relaxes to Ti^(^^3^^)^ during geometry optimization (see Table 1).

**Table 1:** Adsorption energies (*E*_ads_) of isolated Ti atoms on Si(100) of the different sites on the *c*(4 × 2) Si(100) surface

Site	*E*_ads_ [eV atom^−1^]	Site	*E*_ads_ [ eV atom^−1^]
1	−0.06	4	4 → 3
2	2.19	5	0.20
3	0.65	6	−0.14

At site Ti^(^^1^^)^, the Si dimers align and slightly open, increasing the Si–Si bond length from 2.33 Å to 2.63 Å. The Ti atom symmetrically coordinates to neighboring Si atoms, forming four identical bonds of 2.41 Å (see Fig. 1b). In contrast, sites Ti^(^^3^^)^ and Ti^(^^5^^)^ favor tetrahedral coordination, with the ideal geometry hindered by the rigidity of the surrounding surface atoms. Embedding Ti into a perfect tetrahedral arrangement would necessitate breaking or significantly distorting adjacent Si dimers. Such rearrangement is energetically unfavorable, as it would leave opposing Si atoms undercoordinated. As a result, the TiSi_4_ tetrahedron remains distorted at trench sites, with varying degrees of distortion at Ti^(^^3^^)^ and Ti^(^^5^^)^ giving rise to their unfavorable adsorption energies *E*_ads_ (0.65 eV and 0.20 eV). This structural constraint explains why trench sites are less favorable than the top site. Indeed, as will be demonstrated in the following sections, the Ti adsorption energetics are intrinsically linked to the opening of Si dimers.

Furthermore, without considering any potential barriers for reaching this site, Ti can also submerge beneath a dimer (site 6, Fig. 1c), adopting a relatively unperturbed tetrahedral environment. The Ti–Si bond lengths range from 2.45 Å to 2.51 Å, with bond angles spanning from 81° to 121°. The overlying Si dimer is stretched (*r*_SiSi_ = 3.25 Å) and slightly elevated above the surface (Δ*z* = 0.13 Å). However, no extrusion of surface atoms occurs in this scenario, in contrast with findings by Yu *et al.*^46^ and observations made in the context of silicidation of other transition metals.^59^ From an energetic point of view, further embedding of Ti into the Si slab does not occur, as the formation energy of Ti interstitials in bulk Si is highly unfavorable compared to subsurface adsorption layers (see SI, Section 2). These observations align closely with previous studies^46–48^ in terms of geometric relaxations and adsorption energetics. Additionally, these earlier works demonstrated that Ti readily migrates across the surface. Based on these findings, the Ti^(^^1^^)^ site will be preferentially occupied at low coverages, providing the starting point for subsequent growth of the first monolayer.

### 2.2 Interaction between adsorbed Ti pairs

**Fig. 2:**
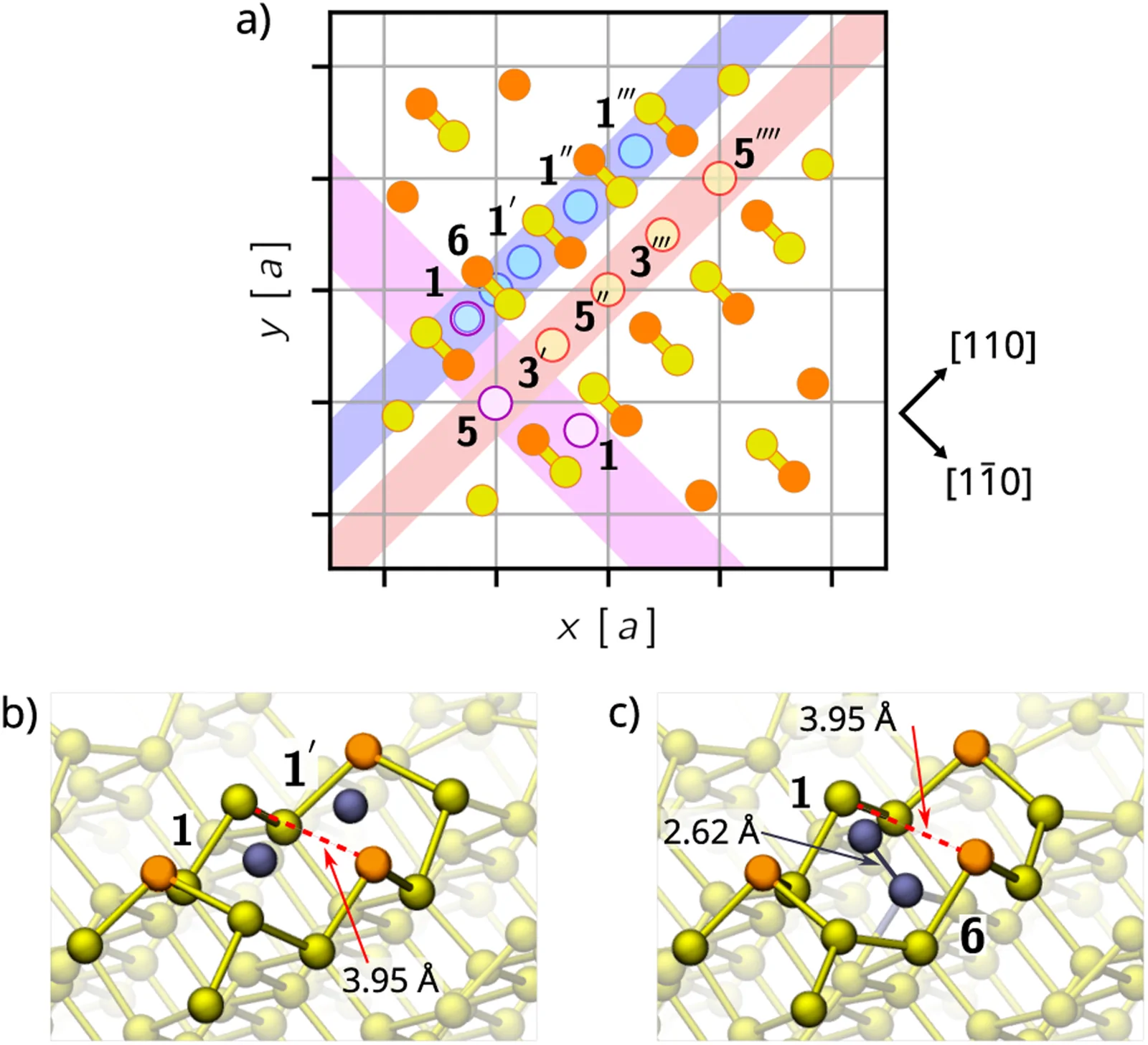
(a) Sketch of the *c*(4 × 2) Si(100) surface to illustrate the direction-dependent pairwise arrangement of Ti adsorbates with the sites on the Si dimer ridge being colored blue, those in the trench being red, and those across along the [11̄0]-direction being purple. The colored stripes are added to guide the eye. Subsurface Si atoms are omitted for visibility. (b) and (c) Depiction of two Ti atoms at position (b) Ti^(^^1^^)^–Ti^(^^1^^′)^ and (c) Ti^(^^1^^)^–Ti^(^^6^^)^, respectively, where Si is colored yellow, and Ti is blue.

To confirm that synergistic effects do not alter the preference for the Ti^(^^1^^)^ site, we now first investigate potential stabilization of the other sites *via* adsorbate pair formation. To systematically explore these interactions, they are categorized based on their orientation: along the [110]-direction on the Si dimer ridges, along the [110]-direction parallel to the trenches between the Si dimer ridges, and along the [11̄0]-direction across the ridges and trenches (see Fig. 2a).

When a second Ti atom (Ti^(^^1^^′)^) adsorbs along the same dimer ridge at closest possible distance, both Ti atoms bind simultaneously to the Si atoms of the shared surface dimer in between. As a result, the dimer reconstruction locally lifts, fully opening the Si–Si bond to 3.95 Å, matching the spacing found on the unreconstructed Si(100) surface (see Fig. 2b). This lateral interaction slightly disfavors co-adsorption as indicated by an association energy *E*_assc_ = 0.04 eV (Table 2). In contrast, for the second and third nearest neighbor configurations (Ti^(^^1″^^)^, Ti^(^^1^^‴)^), each Ti atom independently relaxes the local environment, nearly identical to isolated defects. However, minor residual interactions remain, reflected in small but positive association energies of 0.02 eV and 0.11 eV, respectively.

The adsorption and association energies (*E*_ads_, *E*_assc_) of 2 × Ti@Si(100). The sampling of the Ti pairs has been done in accordance with the symmetry of the Si surface leading to three sets of pairs: on the Si dimer ridge in [110] direction (1–1^*x*^), along the trench in [110]-direction (5-3/5^*x*^), and across along the [11̄0]-direction (1-1/5^*x*^). Compare also Fig. 2a. First neighbors to the reference atom, second neighbors, and so on are denoted as ′, ″, …

**Table:** 

1 [110]	*E*_ads_ [eV atom^−1^]	*E*_assc_ [eV]
1′	−0.04	0.04
1″	−0.05	0.02
1‴	−0.01	0.11
6	−0.18	−0.16

**Table:** 

5 [110]	*E*_ads_ [eV atom^−1^]	*E*_assc_ [eV]
3′	0.20	−0.45
5″	0.28	0.15
3‴	0.55	0.24
5⁗	0.26	0.11

**Table:** 

1 [11̄0]	*E*_ads_ [eV atom^−1^]	*E*_assc_ [eV]
5′	0.12	0.10
1″	−0.05	0.02

Similarly, Ti adsorbates at trench sites exhibit the same general trend: their pairwise association energies (*E*_assc_) are positive, meaning additional Ti occupation in the vicinity of the first adsorbate further reduces the accessibility of these already unfavorable positions. An exception occurs for the directly neighboring Ti^(^^3^^)^–Ti^(^^5^^)^ pairs, for which *E*_assc_ is −0.45 eV. However, even this negative *E*_assc_ is insufficient to offset the intrinsically high *E*_ads_ values of these isolated defects (Table 2), resulting in a net adsorption energy of 0.20 eV atom^−1^. Likewise, an adjacent top-site adsorbate at position Ti^(^^1^^)^ does not provide stabilization when paired with site Ti^(^^5^^)^, as indicated by an *E*_assc_ of 0.10 eV. In this configuration, the presence of Ti at Ti^(^^1^^)^ restricts the flexibility of the adjacent Si dimer, hindering the formation of an optimal coordination environment at Ti^(^^5^^)^. Consequently, clustering of Ti adsorbates at any symmetry site is energetically disfavored due to mutual interference as each atom seeks a high coordination number. The associated energetic penalty for neighboring Ti atoms aligns with values reported previously,^47^ providing a driving force toward the uniform initial growth of the titanium layer.

However, there is one notable exception to the trend discussed above: the interaction between sites Ti^(^^1^^)^ and Ti^(^^6^^)^. This pair is highly favorable, with *E*_ads_ = −0.18 eV atom^−1^ and *E*_assc_ = −0.16 eV, due to the formation of a short Ti–Ti bond measuring 2.62 Å (see Fig. 2c). While the coordination of Ti^(^^6^^)^ remains tetrahedral, similar to the isolated defect, the Ti^(^^1^^)^ site abandons its previously described *D*_4h_-like coordination and moves closer to the neighboring Ti atom. The Si–Si dimer above Ti^(^^6^^)^ remains fully opened, with Si atoms effectively returning to the unreconstructed configuration. This result implies that there exists a stable and energetically favorable local configuration where the pristine Si(100) surface reconstruction is locally reversed. As discussed in detail in the following section, the surface progressively transforms when the number of Ti^(^^1^^)^–Ti^(^^6^^)^ pairs increases, eliminating the initial distinction between trench and dimer-ridge sites.

### 2.3 Increasing coverages

Higher coverages and the resulting combinatorial sampling problem have been approached by accounting all symmetrically unique Ti_ads_ configurations within the third neighbor sphere in accordance with the previously characterized lateral interactions. Expanding these finite-sized configurations to the 4 × 4 slab results in step-wise coverage increases of 0.25 MLs (= 8 atoms), see Methods for more details.

**Fig. 3:**
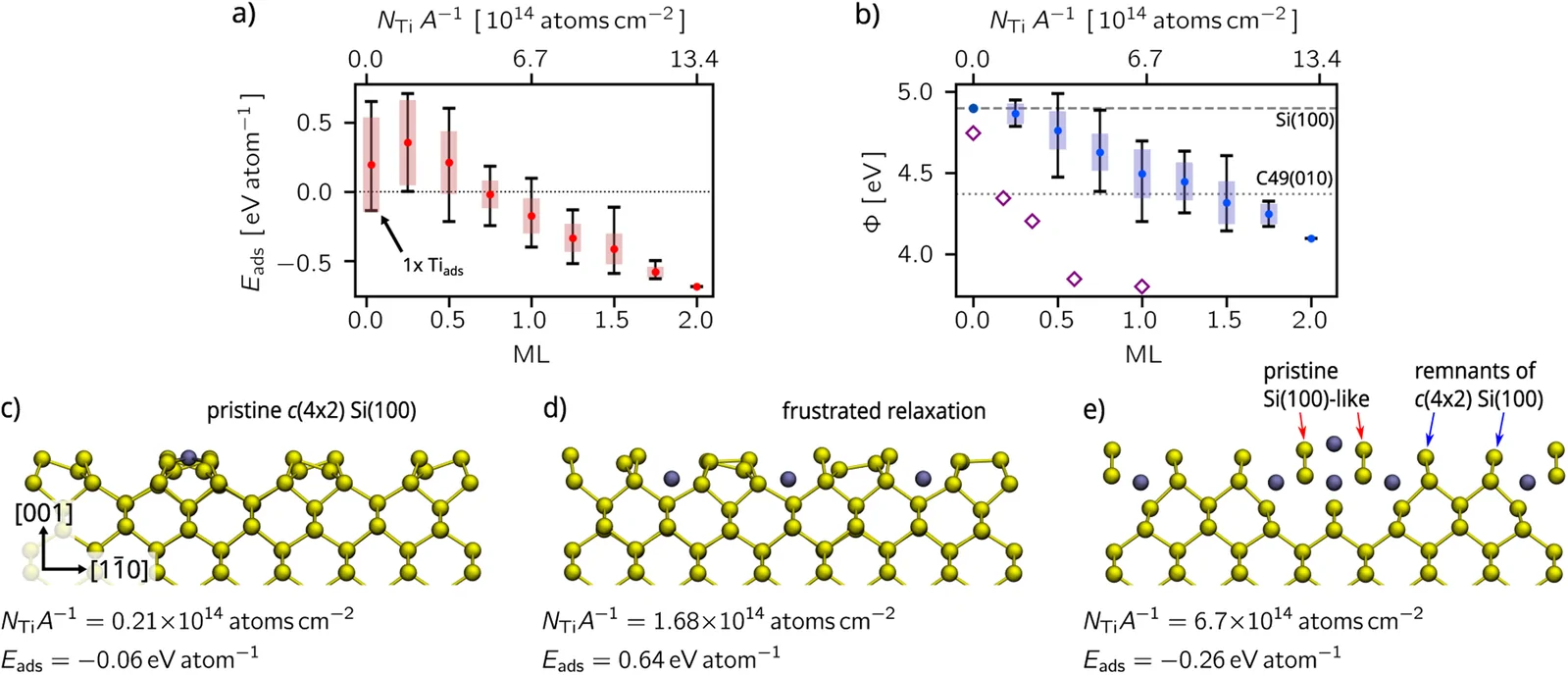
(a) Adsorption energy (*E*_ads_) and (b) the work function *Φ* as a function of the Ti coverage. The adsorbate coverage is given in monolayers (ML) defined *via* the number of adsorbates per surface unit cell and in atoms per surface area (*N*_Ti_*A*^−1^). The error bars indicate the highest/lowest *E*_ads_ and the boxes are the standard deviation of *E*_ads_ of the ensemble of configurations. For comparison, the *Φ* of pristine Si(100) and C49-TiSi_2_(010) is added as dashed and dotted line, respectively. The hollow diamonds are UPS data measured by Taubenblatt and Helms.^60^ (c)–(e) Three representative examples of the slab in (c) the dilute limit, (d) at low concentration, and (e) at medium coverage (from left to right). All slabs are displayed along the [110]-axis.

When increasing the number of Ti adsorbates, a pronounced non-monotonic dependence of the average adsorption energy per adsorbate on coverage emerges, see Fig. 3a. At low coverages, most configurations yield an unfavorable *E*_ads_, peaking at 0.25 monolayers (MLs) with on average 0.35 eV atom^−1^ and a maximum outlier reaching up to 0.71 eV atom^−1^. This behavior can be understood by the interplay of isolated and paired adsorbates described above: at low coverages, *E*_ads_ is strongly influenced by the number of Ti atoms occupying trench sites and the associated pair-wise interactions, and whether the Si dimers are opened in a stable or unstable fashion. Low adsorbate concentrations allow for many unfavorable configurations simply by combinatorics, and the disadvantageous effects of Ti pairs identified in the previous section can amplify one another. An example for such a configuration is displayed in Fig. 3d, where eight Ti^(^^3^^)^ and Ti^(^^5^^)^ atoms collectively attempt to pull neighboring buckled Si dimers to achieve higher coordination. However, the lack of adatoms between the dimers prevents this relaxation, resulting in an overall frustrated geometry (compare also the pristine dimers in Fig. 3c). The observed spread in *E*_ads_ across all coverages is therefore a result of the complex interplay of opened Si dimers with or without stabilization by neighboring Ti atoms, nearest-neighbor effects as discussed in the previous section, and the occupation of trench sites prior to the surface reconstruction being lifted. While these contributions cannot be easily separated, highly ordered configurations support this interpretation, *e.g.*, exclusive occupation of trench sites yields highly unfavorable *E*_ads_ (0.25 ML, *E*_ads_ = 0.71 eV atom^−1^), while perfectly evenly distributed Ti^(^^1^^)^ adsorbates with maximized pair distances yields moderate values (0.25 ML, *E*_ads_ = −0.08 eV atom^−1^).

At coverages above 0.75 ML, *E*_ads_ rapidly becomes negative due to the increasing number of Ti^(^^1^^)^–Ti^(^^6^^)^ pairs, which yield more favorable nearest-neighbor interactions (compare Fig. 2c). More importantly, these pairs locally lift the *c*(4 × 2) Si(100) reconstruction and stabilize the Si atoms in a configuration that is identical to the unrelaxed Si(100) surface, thereby eliminating the distinction between Si dimer ridge and trench sites. As a result, the previously unfavorable Ti^(^^3^^)^, Ti^(^^5^^)^ sites become equivalent to the more favorable Ti^(^^1^^)^, Ti^(^^6^^)^ sites. This is exemplified in Fig. 3e: a stack of Ti^(^^1^^)^–Ti^(^^6^^)^ pairs in the center opens the surface dimers and pushes the adjacent Si atoms back into their original crystallographic positions. This, in turn, stabilizes neighboring Ti adsorbates and enables further Ti deposition on the 4-fold hollow sites between the leveled dimers. Since this effect is local, remnants of the *c*(4 × 2) Si(100) reconstruction are still discernible in the second-nearest row. Eventually, at coverages close to 2 ML, the surface reconstruction is fully reversed and consists only of Ti^(^^1^^)^, Ti^(^^6^^)^ configurations. This transformation reduces the adsorption energies to −0.69 eV atom^−1^ at full occupation of all Ti^(^^1^^)^, Ti^(^^4^^)^ sites.

This has several important consequences. First, successive occupation of Ti^(^^1^^)^–Ti^(^^6^^)^ pairs implies that surface and subsurface sites fill concurrently, even in the dilute limit. A TiSi bilayer therefore nucleates concomitantly rather than only after completing a well-defined Ti monolayer. The calculated work function (*Φ*) decreases smoothly from 4.87 eV for pristine Si(100) to 4.10 eV for the completed TiSi bilayer. These values compare closely with UPS measurements by Taubenblatt and Helms (4.76 eV and 3.80 eV), with absolute deviations of 0.11 eV (bare) and 0.30 eV (bilayer), *i.e.* ≈ 2.3% to 7.9% relative to experiment.^60^ UPS probes macroscopic samples with steps, partial reconstructions, varying termination, thermal disorder, and spatially varying Ti coverages. Our calculations on the other hand are performed on a perfectly ordered slab, which rationalizes these small relative differences.

More importantly, the formation of the TiSi bilayer is a self-promoting process. The gradual opening of Si dimers and pairing Ti^(^^1^^)^–Ti^(^^6^^)^ sites also gradually lifts the surface reconstruction, eliminating the distinction between trench and ridge site and leaving only the energetically favorable Ti^(^^1^^)^, Ti^(^^6^^)^ sites. Consequently, each Ti^(^^1^^)^–Ti^(^^6^^)^ pair creates additional Ti^(^^1^^)^, Ti^(^^6^^)^ sites, driving a positive feedback loop where Ti adsorption becomes increasingly favorable with coverage. Thus, the deposition of Ti and the initial formation of a mixed TiSi layer is not solely explained by adsorption energies of individual atoms alone, but by the complex interplay of Ti–Ti pairing and opening of the Si surface dimers. Ultimately, the structure of the TiSi bilayer is dictated by how the Ti^(^^1^^)^–Ti^(^^6^^)^ pairs are embedded into the Si(100) surface.

### 2.4 Characterization of the TiSi bilayer

**Fig. 4:**
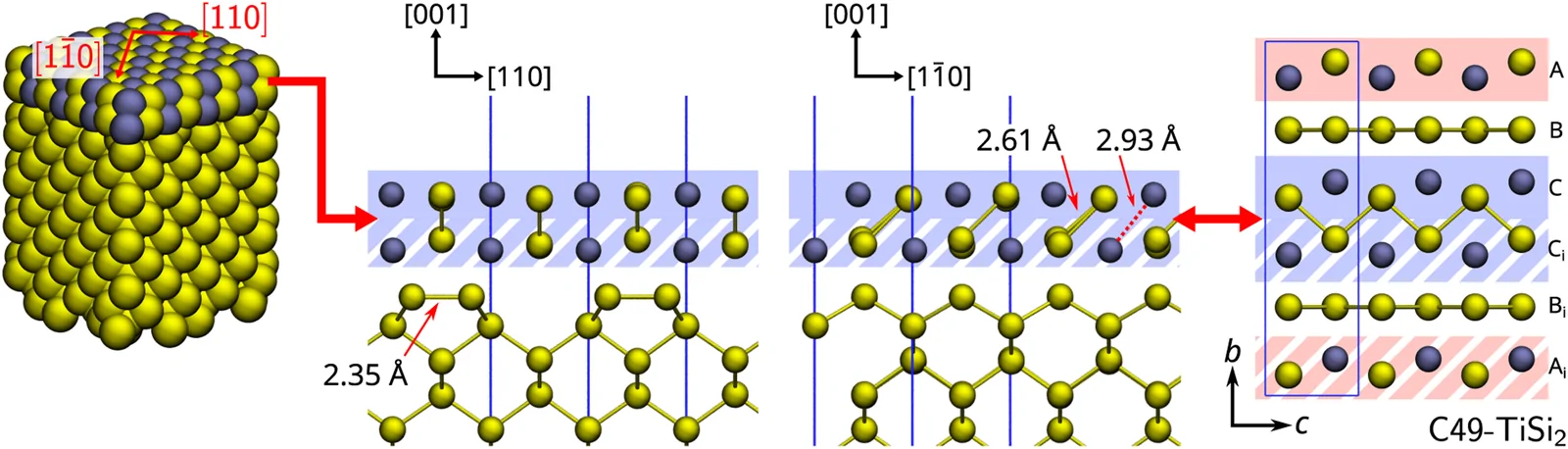
Depiction of a slab covered by two monolayers of Ti, with close-up views of the bilayer along the [110] and [11̄0] directions, together with a 3 × 1 × 3C49-TiSi_2_ cell for comparison. The blue lines outline the 1 × 1 Si(100) surface unit cell.

The fully occupied TiSi bilayer consists of alternating zigzag-shaped Ti–Ti and Si–Si chains oriented along the [110] lateral direction in the Si(001) surface (see Fig. 4). This arrangement yields local coordination environments that differ markedly from the *T*_d_ motif in α-Si and the *D*_3h_ motif in α-Ti. The Si–Si bonds are elongated to *r*_SiSi_ = 2.61 ± 0.12 Å relative to their equilibrium length of 2.35 Å in α-Si,^61^ while the Si–Si–Si angles are compressed to *θ*_Si_ = 95.28 ± 2.75°. Minor out-of-plane corrugations in the bilayer lead to small variations in bond lengths and angles (see Table 3). It is also noteworthy that the underlying Si interface layer reconstructs to form Si–Si dimers similar to those on the Si(100) with the dimer buckling being lifted due to stabilizing interactions with the TiSi bilayer above.

**Table 3:** Bond lengths (*r*), angles (*θ*), and Bader charges (*q*_bader_) in the TiSi bilayer on the Si(100) surface, in the AA_i_ layer of bulk C49-TiSi_2_, and in crystalline α-Si and α-Ti, respectively. All data are from DFT calculations at the PBE level

		TiSi bilayer	C49-TiSi_2_	α-Si/α-Ti
*r*_Ti–Ti_	Å	2.93 ± 0.15	3.32	2.92
*r*_Si–Si_	Å	2.61 ± 0.12	2.40	2.36
*θ*_Ti_	deg	82.29 ± 1.03	65.02	60
*θ*_Si_	deg	95.28 ± 2.75	96.27	109.47
*q*_bader_(Ti)	*e*	0.96	1.07	—
*q*_bader_(Si_A/C_)	*e*	−0.82	−0.53	—

The comparison to TiSi_*x*_ polymorphs from the literature with the TiSi bilayer reveals a remarkable structural similarity to the C49-TiSi_2_ phase, particularly to the AA_i_ and CC_i_ layers (see Fig. 4). Note that the AA_i_ layer shown in Fig. 4 is symmetrically equivalent to the CC_i_ layer; a more detailed description of the C49-TiSi_2_ phase is provided in the SI, Section 1.3. Most strikingly, both the TiSi bilayer and the C49 structure feature zigzag chains of Ti and Si atoms. Such a motif is absent in other TiSi_*x*_ polymorphs, especially in the competing C54 phase, where the Ti atoms are spaced out more evenly and thus do not align with the Ti-rich TiSi layer. Hence, the resulting structure suggests that the TiSi bilayer acts as a template for the nucleation of C49-TiSi_2_, while the formation of C54-like structures would require more drastic intermixing, which is unlikely due to the low diffusivity of Ti interstitials^62^ and the rapid increase in defect formation energy beneath the surface.

Expressed in the C49 lattice basis, the C49(010) plane lies parallel to the Si(100) plane, with the lattice vectors a_C49_ and c_C49_ rotated by 45° with respect to the silicon lattice. The resulting lattice mismatch is 4.18%. The diagonal of the Si a_Si_b_Si_-plane is 7.73 Å, slightly larger than twice the C49 lattice constants *a* and *c* (each 3.71 Å at the PBE level). Hence, the TiSi bilayer combines a distinctive structural motif with a favorable crystallographic alignment, supporting its role as a nucleation seed for C49-TiSi_2_ growth.

In the broader context of C49-TiSi_2_ growth, defect formation energies (*E*_form_) are critical, as defects and atomic diffusion have been identified as key factors favoring C49-TiSi_2_ over C54-TiSi_2_. Experimental studies consistently show that Si diffuses more rapidly than Ti, thereby promoting crystal growth.^41,63–66^ During annealing, the TiSi_*x*_ layer thus extends deeper into the silicon bulk, while excess Si forms a surface layer.^60^ To better understand this process, Brown *et al.*^44^ computed bulk defect energetics for both polymorphs, reporting that Si and Ti vacancies are roughly 1 eV to 1.5 eV more favorable in C49 than in C54. We have repeated those calculations for bulk C49 within our computational setup and find that the defect formation energies remain positive with *E*_form_(*V*_Si_^bulk^) = 1.07 eV and *E*_form_(*V*_Ti_^bulk^) = 2.99 eV, see Table 4.

**Table 4:** Defect formation energies *E*_form_ of titanium antisites (Ti_Si_), silicon antisites (Si_Ti_), titanium vacancies (*V*_Ti_), and silicon vacancies (*V*_Si_) in the TiSi bilayer. For comparison, *E*_form_ in bulk C49-TiSi_2_ is included. All energies are in eV

Defect	Layer		C49-TiSi_2_	
	Top	Subsurface	A/C	B
Ti_Si_	0.77	−0.68	0.41	0.88
Si_Ti_	1.13	1.52	4.49	—
V_Ti_	0.75	1.65	2.99	—
V_Si_	−0.05	−0.12	1.07	1.37

In contrast, the TiSi bilayer exhibits much lower defect formation energies, with Si vacancies even becoming energetically favorable (−0.05 eV and −0.12 eV; see Table 4). Similarly, *E*_form_(*V*_Ti_) drops to 0.75 eV, significantly below the bulk value of 2.99 eV. We attribute this reduction to strain induced by lattice mismatch and surface effects, which vacancies help to relieve. The TiSi bilayer thus provides an accessible pathway for atom migration, consistent with experimental observations.

### 2.5 Discussion

In the context of Ti growth on Si, our results indicate that initial deposition first populates adsorption sites along the dimer ridges: any nearest-neighbor pairing carries an energetic penalty because it forces the opening of a surface dimer. The decisive step for further growth is the accumulation of a fraction of Ti atoms into the first subsurface layer, which locally lifts the surface reconstruction and renders previously unfavorable adsorption sites accessible. Although the minimum energy path for submerging Ti is not explicitly calculated here, the observation of minor intermixing of Ti and Si in the order of 1 to 3 monolayers prior to annealing suggests that Ti^(^^1^^)^–Ti^(^^6^^)^ pairs eventually form.^35,67,68^ This is consistent with the NEB calculations of Briquet *et al.*,^47^ who reported migration barriers of 1.0 eV to 1.5 eV for isolated Ti atoms between the various adsorption sites. Notably, the lowest barrier for diffusion into the subsurface is in the same energy range with only 0.88 eV, indicating that subsurface diffusion can readily occur at elevated temperatures.^47^ Given that Ti is a slow diffuser in Si,^62^ this process is potentially aided by the dynamic flip-flop motion of surface dimers, which undergo a continuous transition between the *p*(2 × 2) and *c*(4 × 2) surfaces already at low temperatures.^56,58,69–72^ Nevertheless, determining the adsorption kinetics remains a question for future research.

Once the TiSi bilayer is complete, the growth mode changes abruptly. Additional Ti adsorbates bind much more weakly to the TiSi surface (*E*_ads_ = 1.43 eV), whereas Ti–Ti interactions outweigh surface binding (*E*_assc_(2 × Ti@TiSi) = −0.47 eV; see SI, Section 3). This promotes Ti clustering irrespective of the substrate's local orientation, consistent with Stranski–Krastanov growth,^33–36^ in which a uniform wetting layer is followed by island formation. Compared with earlier computational models,^47^ our results indicate a clear-cut switch in growth mode. Importantly, weakly attractive association energies at low Ti coverage do not necessarily signal the onset of clustering; they can also reflect local cooperative effects. The abrupt change in adsorption behavior once the bilayer is complete rather points to a transition driven by the reduced surface affinity of Ti for the TiSi surface.

These findings suggest a practical route to mitigate the undesired formation of C49-TiSi_2_. The TiSi bilayer on Si(100) constitutes an epitaxial template: its zigzag Ti–Si chains reproduce the A/C layers of C49, with C49(010)‖Si(100), a_C49_ and c_C49_ rotated by 45°, and only 4.18% lattice mismatch. Accordingly, our model predicts out-of-plane growth along C49[010], consistent with experiment.^73^ Reports of alternative orientations under rapid thermal annealing (up to 800 °C)^74–76^ and the identification of C49(131) as the lowest-energy surface^77^ indicate that at elevated temperatures kinetics (vacancy formation, interfacial strain relaxation, and surface migration) can override this epitaxial preference.

If Si(100) seeds C49-TiSi_2_*via* this template, then breaking the surface structure before annealing should suppress C49-TiSi_2_, because if the ridge-trench morphology is lost, the driving force for the anisotropic embedding of Ti along the [110] direction disappears as well. In the absence of directionality, the TiSi_2_ growth is then governed by thermodynamic phase stability, favoring the formation of C54-TiSi_2_. Consistent with this mechanism, it has been observed that preamorphization of Si(100) promotes C54-TiSi_2_ ^18,21,78–82^ provided it precedes the TiSi_*x*_ formation step.^43^ Inserting thin refractory interlayers (Mo, Ta, Nb, W) yields similar outcomes,^83–88^ either through blocking adsorption sites and disruption the Ti deposition pattern, or through forming a mixed MSi_*x*_ phase^62,89,90^ thereby altering the adsorption energetics. Thus, any perturbation to the Si(100) surface would result in suppressing the surface-topology–directed nucleation of C49-TiSi_2_.

More broadly, these findings shed new light on the design principles governing metal-silicon interfaces in nanoelectronic applications. In the present case, the formation of C49-TiSi_2_ is facilitated by the spatial arrangement of the Si–Si dimers and the favorable incorporation of Ti into the reconstructed surface. This raises the question whether the adsorption behavior of other transition metals used in MS junctions is likewise influenced by the Si(100) surface reconstruction, particularly for elements with similar atomic radii and bonding characteristics. Although such element-specific adsorption requires dedicated modeling, this study demonstrates how understanding the unique chemistry of *c*(4 × 2) Si(100) can provide a route to controlling nucleation and interface growth through targeted surface preparation. As such, these new insights could help to selectively grow metal silicide polymorphs, thereby enabling the integration of a broader range of materials in temperature-sensitive material stacks and ultimately opening up new opportunities to further optimize device performance.

## 3 Conclusions

In summary, we have comprehensively modeled the adsorption of Ti on *c*(4 × 2) Si(100), from isolated defects in the dilute limit up to coverages corresponding to two monolayers. For isolated Ti, the preferred adsorption site is in the fourfold hollow site between two adjacent Si surface dimers rather than in the trenches between them. The interactions between Ti adsorbates are mostly repulsive, giving rise to a uniform initial growth of TiSi_*x*_; yet Ti interstitials in the first subsurface layer form a strong bond with Ti adsorbates on the surface. These Ti–Ti pairs disrupt the Si dimers and locally lift the surface reconstruction. As a result, the trench adsorption sites become symmetrically equivalent to the favorable adsorption sites, thereby creating a self-promoting mechanism in which higher coverage leads to the formation of more favorable sites and Ti–Ti pairs.

The resulting TiSi bilayer reproduces characteristic low-symmetry motifs of the C49-TiSi_2_ structure that are unique to this particular polymorph. This suggests that the initial formation of TiSi_2_ is governed primarily by the topology of the reconstructed Si(100) surface: the arrangement of buckled Si dimers dictates the initial adsorption, while the spacing between the crystallographic sites in Si is just the right size to embed Ti atoms in between. Eventually, the initial Ti adatom pairs in conjunction with the opening of the Si dimers guide the adsorption process towards the characteristic bilayer structure.

The presented model provides a unified explanation for the initial formation of a Ti rich TiSi_*x*_ layer at low temperatures, the Stranski–Krastanov growth mode, and offers a plausible mechanism by which the Si(100) surface serves as the nucleation template for C49-TiSi_2_, despite its thermodynamic disadvantage relative to C54-TiSi_2_. This interpretation also aligns with experimental findings that disrupting the surface structure through preamorphization or refractory metals inhibits the C49 formation. However, our model only includes the first two monolayers and therefore cannot establish a fully quantifiable link. Resolving this will require future studies that extend to higher Ti exposure to model the interface of thicker C49-TiSi_2_ films on Si(100). Nevertheless, this novel understanding of the unique growth mechanism of TiSi_2_ aids the targeted design of smooth metal-semiconductor junctions to pave the way for next-generation 3D-architectures and photocatalysts.

## 4 Computational methods

All DFT calculations were performed spin-polarized using CP2K^92,93^ employing the PBE exchange-correlation functional^94,95^ with the DZVP-MOLOPT basis set^96^ and the GTH pseudopotentials^97,98^ to describe the core electrons. The plane-wave cut-off and relative cut-off were converged to 700 Ry and 80 Ry ensuring the SCF convergence to an accuracy of 1 × 10^−7^ eV, including only the *Γ*-point for Brillouin zone sampling. Fermi–Dirac smearing with an electronic temperature of 100 K was applied to aid SCF stability during the transition from semiconducting Si to metallic TiSi_*x*_. Bader charges were calculated using the code developed by the Henkelman group.^99–103^

**Fig. 5:**
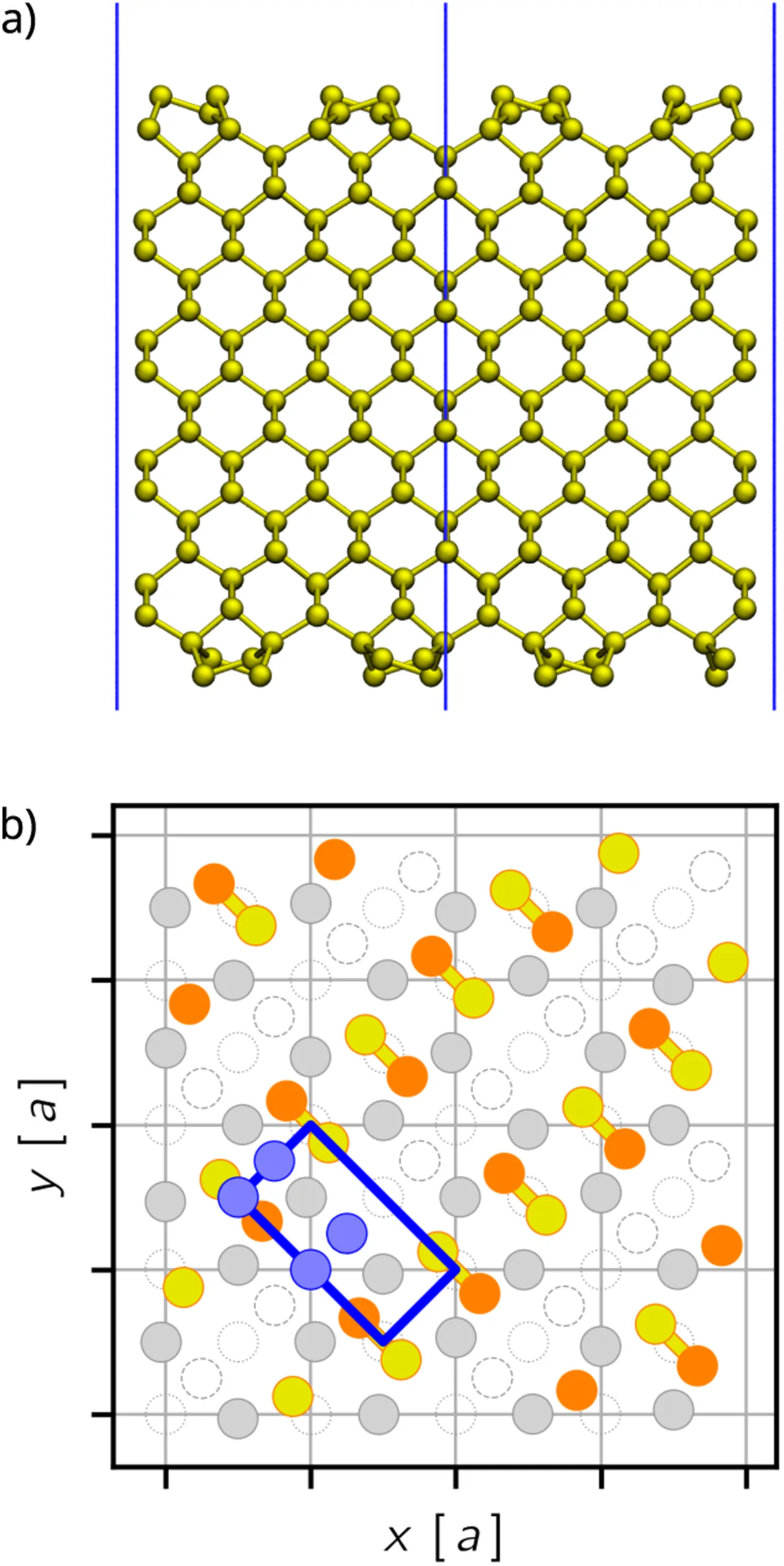
(a) The *c*(4 × 2) Si(100) slab viewed from the [110]-direction (b) schematic top view of the *c*(4 × 2) Si(100) slab with the adsorption sites and the reduced cell used for the exhaustive sampling with icet code.^91^

All Si slab models were prepared using the ASE^104^ package based on bulk lattice constants from a fully optimized 3D periodic 3 × 3 × 3 cell. The silicon slabs were converged to the surface energy *E*_surf_ < 0.3 eV Å^−2^ requiring a thickness of 20 atomic layers (10 crystallographic layers) and lateral expansion of 4 × 4 in the [100]- and [010]-directions leading to a total of 640 atoms. A vacuum spacing of 40 Å was introduced along the surface normal ([001]-direction). The *c*(4 × 2) Si(100) surface reconstruction was chosen as the basis for all adsorption configurations as this is the lowest-energy surface configuration according to literature^57,58^ and our benchmark (see Fig. 5a, SI Section 1). Configurations with more than two adatoms were systematically enumerated using the icet code^91^ with a reduced cell for the adsorbates in accordance with the isolated adsorbates as presented in Section 3.1 below. The reduced cell displayed in Fig. 5b is expanded up to an interaction range of 2, thereby capturing all relevant next-nearest-neighbor interactions as determined in Section 3.2. Structures were visualized using VMD.^105^

The average adsorption energies per adsorbate (*E*_ads_) are calculated *via*1
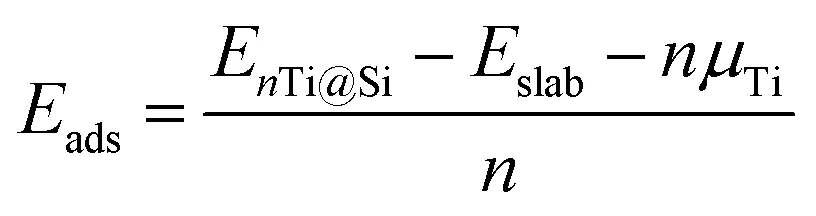
where *µ*_Ti_ is the chemical potential of Ti, referenced to bulk α-Ti. While earlier works^47,48^ used isolated Ti atoms in vacuum for *µ*_Ti_ to approximate conditions of atomic layer deposition (ALD), we adopt the bulk reference (*µ*_Ti_ = *E*_bulk_(α-Ti)/*N*_Ti_) to better assess the competition between Ti–Si binding and Ti–Ti clustering. As a result, our reported *E*_ads_ values are shifted by the cohesive energy of bulk α-Ti, approximately −5.27 eV, relative to the quoted studies. The association energy (*E*_assc_) is defined by comparing *E*_ads_ of a system with *N* adsorbates to the sum of isolated adsorbates:2
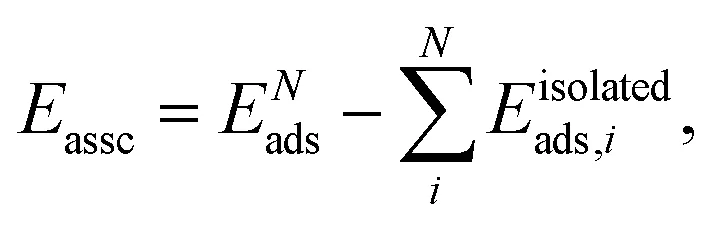
where a negative value indicates an energetic preference for clustering and a positive value repulsion among adsorbates.

## Author contributions

L. H.: conceptualization, data curation, formal analysis, investigation, methodology, software, validation, visualization, writing – original draft. J. C.: conceptualization, supervision, validation, writing – review and editing. J. M.: funding acquisition, resources, validation, writing – review and editing. E. O.: conceptualization, resources, supervision, validation, writing – review and editing.

## Conflicts of interest

There are no conflicts to declare.

## Supplementary Material

NA-008-D6NA00467A-s001

## Data Availability

All structures generated in this study are available *via*zenodo.org at https://doi.org/10.5281/zenodo.18242845. Supplementary information (SI): additional computation details for bulk α-Si, Si(100) surface and bulk C49-TiSi_2_, Ti interstitial defects close to the Si(100) surface, Ti adsorption on the TiSi bilayer. See DOI: https://doi.org/10.1039/d6na00467a.
