# Supplementary material for: Topology-directed silicide formation: an explanation for the growth of C49-TiSi2 on the Si(100) surface
Source: Nanoscale Adv. 2026 Aug 7;8(18):5256–66. doi: 10.1039/d6na00467a (PMC13474482; doi:10.1039/d6na00467a)
Supplement: Supplementary file 1 [file NA-008-D6NA00467A-s001.pdf]

Supplementary Information (SI) for Nanoscale Advances.
This journal is © The Royal Society of Chemistry 2026

Supporting Information: Topology-Directed Silicide Formation: An Explanation for the Growth of C49-$TiSi_2$ on the Si(100) Surface

Lukas Hückmann,[†,‡,¶] Jonathon Cottom,[‡,†] Jörg Meyer,[†] and Emilia Olsson[*,‡]

†*Leiden Institute of Chemistry, Gorlaeus Laboratories, Leiden University, P.O. Box 9502, 2300 RA Leiden, The Netherlands*

‡*Advanced Research Center for Nanolithography, Science Park 106, 1098 XG Amsterdam, The Netherlands*

¶*Institute for Theoretical Physics, University of Amsterdam, Postbus 94485, 1090 GL Amsterdam, The Netherlands*

E-mail: e.olsson@arcnl.nl

Contents

1 Additional Computational Details **S-3**

1.1 Bulk α-Si S-3

1.2 Si(100) Surface S-4

1.3 Bulk C49-$TiSi_2$ S-6

2 Ti Interstitial Defects Close to the Si(100) Surface **S-8**

3 Ti Adsorption on the TiSi Bilayer **S-9**

References **S-11**

1 Additional Computational Details

1.1 Bulk α-Si

At standard conditions, there is only a single silicon modification (α-Si) which crystallizes in a face-centered cubic cell (Fd$\bar{3}$m, No. 227), equivalent to the diamond structure, see Figure S1 and Table S2. The lattice constant obtained from an optimized $4 \times 4 \times 4$ cell is slightly elongated with respect to the experimentally measured value ($> 1\,\%$), resulting in slightly elongated equilibrium bonds. This has been observed previously for PBE[S1] and is well within an acceptable range. Thus, the lattice constant provided in Table S2 are used to construct for modeling all Si(100) slabs.

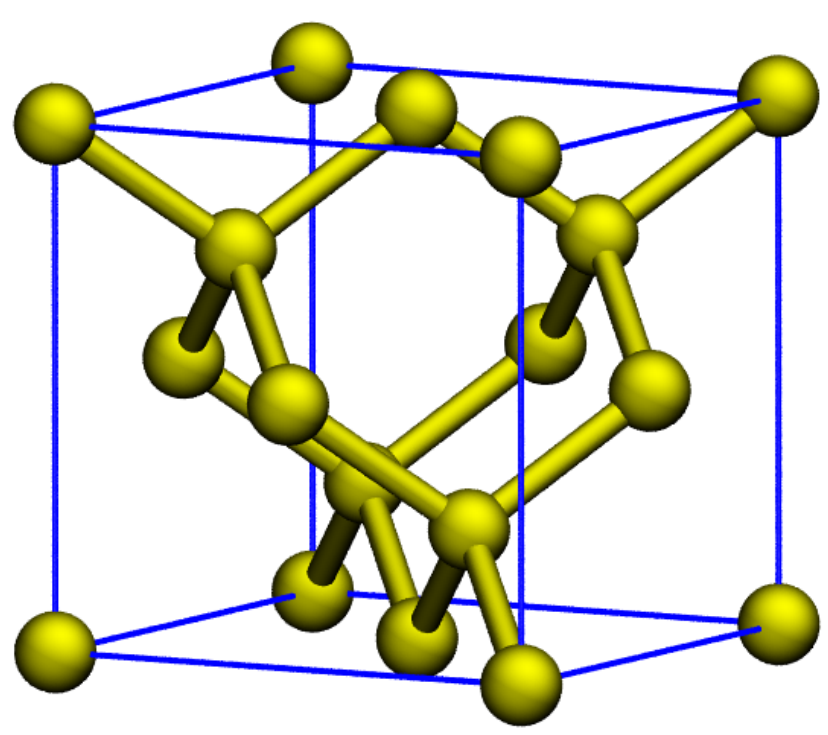

Figure S1: The unit cell of α-Si.[S2]

Table S1: The symmetry of α-Si.

	α-Si
crystal system	cubic
space group	Fd$\bar{3}$m (No.227)
forumla units Z	8

Table S2: Lattice constants and structure of α-Si calculated with CP2K compared to VASP and literature.

	PBE (CP2K)	PBE (VASP)	literature
a / Å	5.4636	5.4685	5.430880(35)[S2]
V / Å^3	160.180	163.533	163.095[S2]
$r_{\mathrm{Si-Si}}$ / Å	2.366	2.368	2.352[S3]
E_{coh} / eV atom^{-1}	4.74	–	4.66[S4,S5]

1.2 Si(100) Surface

α-Si has two relevant surfaces along the (100) and the (111) plane, where the surface energy (E_{surf}) is slightly lower for the (100) surface with $E_{\mathrm{surf}}(100) = 0.0718\,\mathrm{eV\,\AA^{-2}}$ compared to $E_{\mathrm{surf}}(111) = 0.0923\,\mathrm{eV\,\AA^{-2}}$. Both surfaces have been studied extensively and wafers with the respective orientation are commercially available Due to the plethora of experimental literature for the deposition of Ti on Si(100) and their widespread usage in electronic devices, this study focuses on the (100) surface.

Each surface unit cell of the Si(100) surface comprises two-fold-coordinated Si atoms (Figure S2a), which tilt towards each other to form dimers to increasing their coordination number and lowering the E_{surf} (Figure S2b). The dimer formation is well documented in

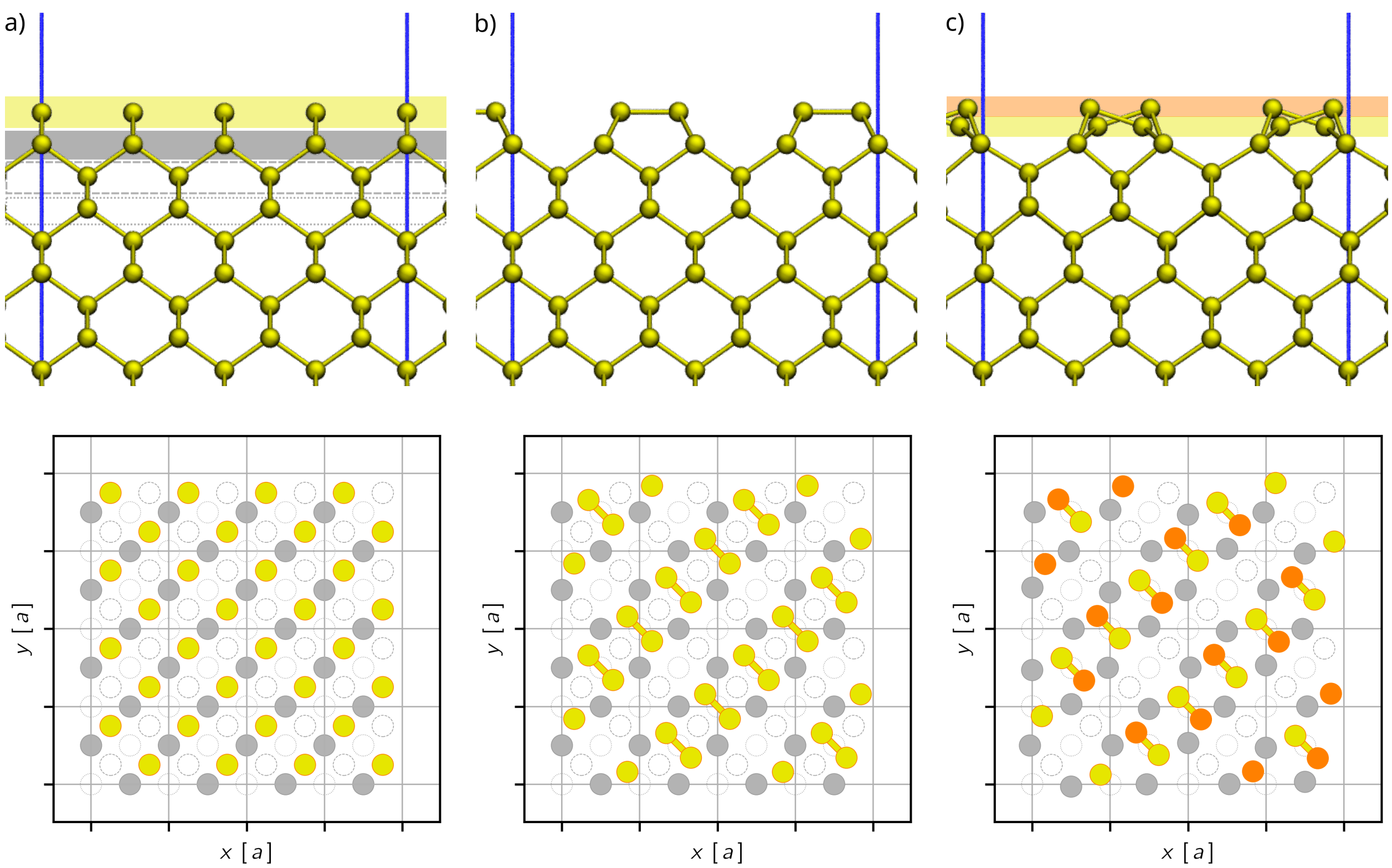

Figure S2: Depiction of the Si(100) surface of a) the unrelaxed surface, b) the surface with Si-Si dimers, and c) the surface with buckled dimers. In the row below there is a sketch with a top-view on the xy-plane of each surface with the yellow circles being the atoms on top, the gray ones being the underneath, followed by the dashed-hollow and dotted-hollow ones. The orange circles in c) represent the buckled dimers; compare the color bars in the top panel. The grid indicates the surface unit cells.

other computational studies and confirmed by STM images.[S6–S8] As has been previously reported[S9,S10] and is reproduced in our calculations, the dimers are not stable and relax further into the "buckled" configuration shown in Figure S2c, where one Si atom sinks into the surface while the other is slightly elevated. That leads to a variety of different dimer patterns at the surface, of which the one shown with parallel dimers with alternating elevation is the lowest energy one ($c(4 \times 2)$ in Wood's notation for overlayer structures). The minimal lateral size for which such a surface termination can be constructed is 4×4, which is why slabs of this size are used throughout this study.

1.3 Bulk C49-$TiSi_2$

Titanium silicide ($TiSi_x$) exhibits a rich phase diagram, of which $TiSi_2$ and Ti_5Si_3 are the most common compositions.[S14] When depositing Ti on Si, it is observed that the C49-$TiSi_2$ polymorph (*Cmcm*, space group 63) is formed first. The unit cell of C49-$TiSi_2$ polymorph is characterized by substantial anisotropy with the *b*-axis being close to four times as large as the *a*- and *c*-axis, see Figure S3a and Table S3. The structure is comprised of three layers A, B, C along the [010]-axis, with an inversion center at $(0.5, 0.5, 0.5)$, such that there are three more layers A_i, B_i, C_i, see Figure S3b,c. Layer B can be described as a square uniform rectangular grid of Si atoms in the *ac*-plane, while the Ti and Si atoms in the layers AA_i and CC_i together form an alternating zig-zag pattern. The AA_i and CC_i layers are symmetrically identical to each other except for being offset by half a unit cell. The Ti chains in the AA_i/CC_i layer are widened with $r_{Ti-Ti} = 3.36$ Å compared to the 2.92 Å found

Table S3: The lattice constants data of C49-$TiSi_2$.

lattice constant	unit	$5 \times 1 \times 5$ PBE/DZVP	R2SCAN/DZVP	literature PBE[S11]	Exp.[S12,S13]
a	Å	3.571	3.540	3.548	3.56
b	Å	13.565	13.482	13.568	13.61
c	Å	3.574	3.552	3.578	3.56

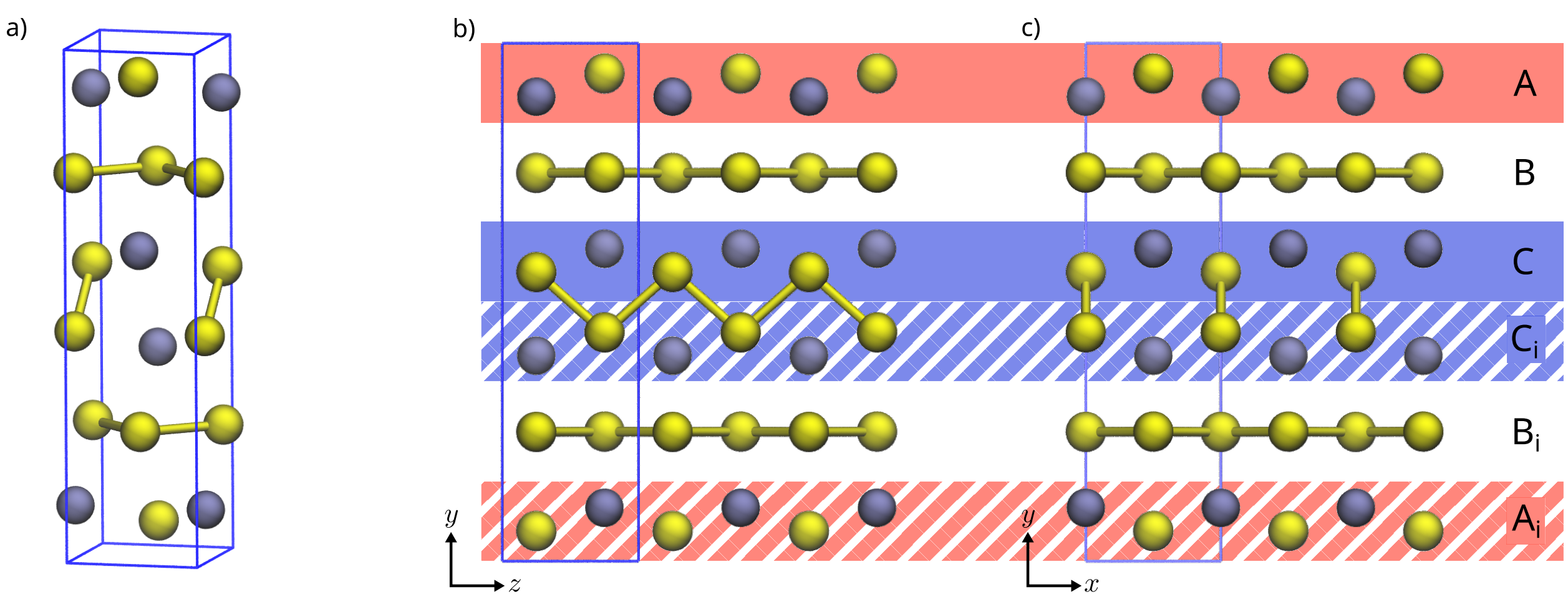

Figure S3: a) Unit cell of C49-$TiSi_2$ b)/c) side view on a $3 \times 1 \times 3$ super cell along the [100] and [001] direction, respectively.

in α-Ti. In contrast, the Si atoms in zig-zag chain are tighter packed with 2.41 Å and a Si−Si−Si angle of 96.89°.

The C49-$TiSi_2$ phase has been studied computationally by Brown *et al.*[S11]. They focused mostly on the bulk and on point defects in the crystal To validate our computational set-up, we reproduced their results, see Tables S3 to S5. The cell parameters and Bader charges are in good agreement, but there is a notable discrepancy in the defect formation energies (E_{form}) to the values by Brown *et al.*[S11], which is probably due to thermal corrections applied. Since the magnitude of E_{form} is similar and our data is consistent across various levels of theory, the deviation is considered acceptable.

Table S4: The Bader charges of Ti and Si in C49-$TiSi_2$. The layer enumeration from Figure S3 applies.

atom	$5 \times 1 \times 5$		Brown et al.[S11]
	PBE/DZVP	R2SCAN/DZVP	PBE
$Si_{A/C}$	−0.50	−0.53	−0.67
Si_B	−0.37	−0.40	−0.43
Ti	0.99	1.07	1.10

Table S5: Defects formation energies.

defect	unit	$5 \times 1 \times 5$		Brown et al.[S11]
		PBE/DZVP	R2SCAN/DZVP	PBE
V_{Ti}	eV	2.990	3.115	1.89
Si_{Ti}	eV	4.490	5.063	3.92
V_{Si}(A/C)	eV	1.066	0.687	0.418
V_{Si}(B)	eV	1.366	1.079	0.633
Ti_{Si}(A/C)	eV	0.413	0.128	0.695
Ti_{Si}(B)	eV	0.880	0.756	1.38

2 Ti Interstitial Defects Close to the Si(100) Surface

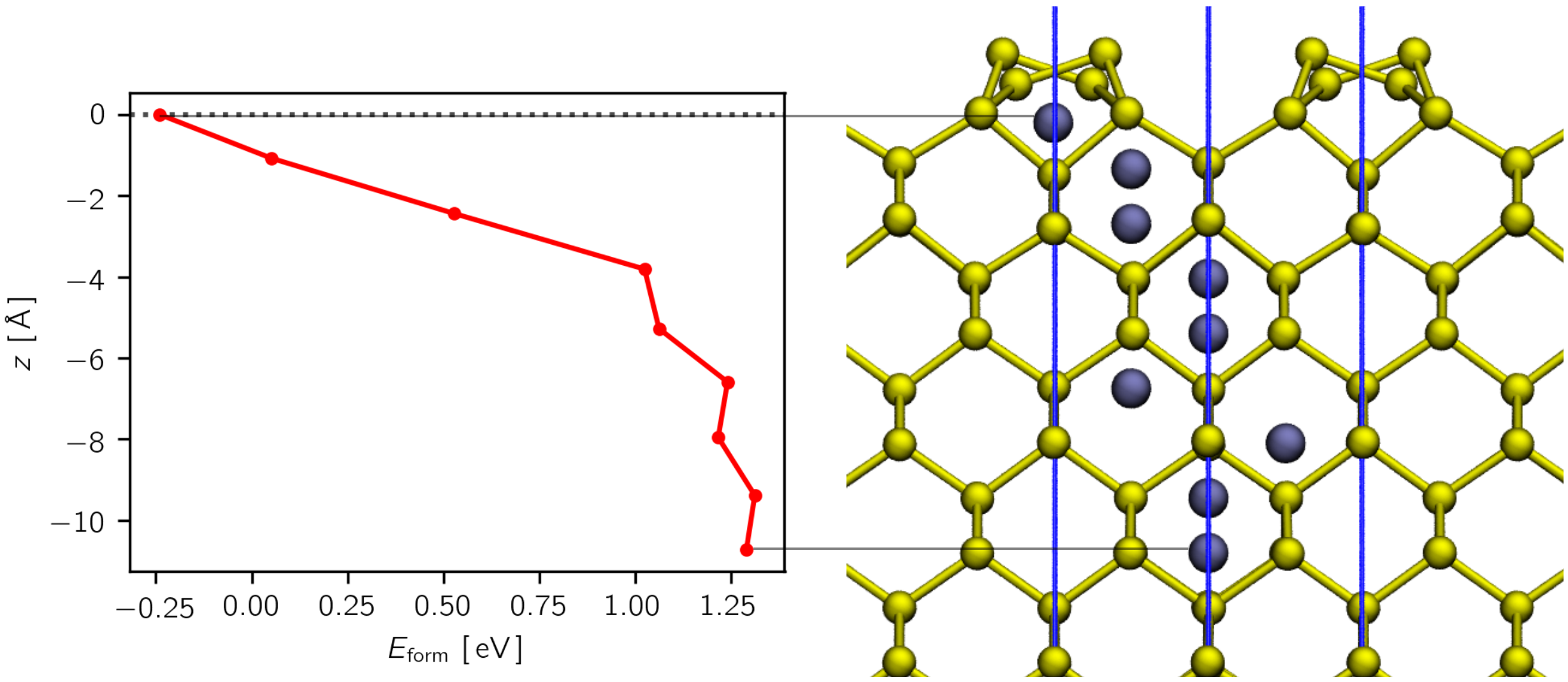

Figure S4: a) The formation energy E_{form} of titanium interstitials depending on the depth z underneath the surface. b) Sketch of $\mathrm{Ti_i}$ positions in the Si crystal. $\mathrm{Ti_i}$ are only stable in the tetrahedral gaps in the FCC structure. The blue lines are the edges of the 1×1 Si unit cell.

3 Ti Adsorption on the TiSi Bilayer

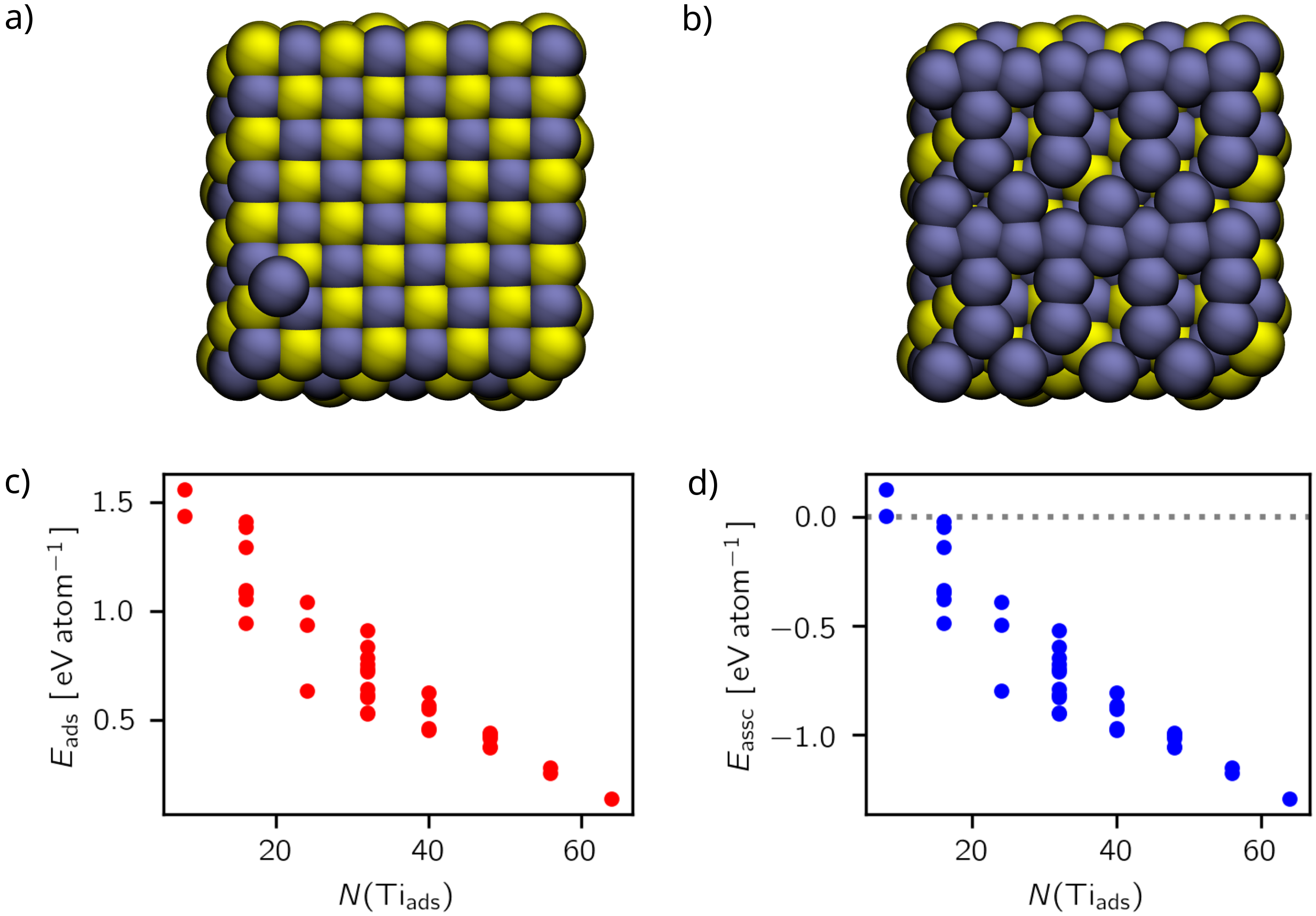

Figure S5: Depiction of a) an isolated Ti adsorbate and b) several Ti adsorbates clustering on the TiSi bilayer. Ti is colored blue-gray, Si is yellow. c) The adsorption energies E_{ads} per atom and d) the association energies E_{assc} as a function of number of adsorbates on the TiSi bilayer.

As illustrated in see Figure S5a, at topview of the TiSi bilayer constitutes a checkerboard pattern of Ti and Si, where the most favorable adsorption site for additional both Ti atoms is at the cavity at the intersection between four surface atoms, The adsorption energy is positive with 1.43 eV, i.e. it is significantly less favorable than the adsorption on the pristine Si(100) surface. At the same time, the association energy E_{assc} shows with -0.47 eV a clear tendency for the Ti adsorbates to cluster. Formation of clusters reminiscent of the α-Ti structure is being facilitated as the Ti coverage increases Figure S5b shows an example where the onset of the hexagonal pattern characteristic of the hcp crystal structure becomes discernible. As a result both E_{ads} and E_{assc} both decrease (see Figure S5c and d). Moreover, the adsorbate

positions become largely independent of the underlying surface and are instead dictated mainly by Ti−Ti interactions, as the symmetry of the cluster does not coincide with the substrate. Hence, the gain in energy is due to the increasing number of Ti−Ti interactions and the stabilization within the clusters while the TiSi bilayer surface remains unaltered.

References

(S1) Sánchez, K.; Aguilera, I.; Palacios, P.; Wahnón, P. Assessment through first-principles calculations of an intermediate-band photovoltaic material based on Ti-implanted silicon: Interstitial versus substitutional origin. *Phys. Rev. B* **2009**, *79*, 165203, DOI: 10.1103/PhysRevB.79.165203.

(S2) Hubbard, C. R.; Swanson, H. E.; Mauer, F. A. A silicon powder diffraction standard reference material. *J. Appl. Crystallogr.* **1975**, *8*, 45–48, DOI: 10.1107/S0021889875009508.

(S3) Wiberg, E.; Wiberg, N.; Holleman, A. F. *Anorganische Chemie*, 103rd ed.; De Gruyter: Berlin and Boston, 2017.

(S4) Davis, S. G.; Anthrop, D. F.; Searcy, A. W. Vapor Pressure of Silicon and the Dissociation Pressure of Silicon Carbide. *J. Chem. Phys.* **1961**, *34*, 659–664, DOI: 10.1063/1.1701004.

(S5) Farid, B.; Godby, R. W. Cohesive energies of crystals. *Phys. Rev. B* **1991**, *43*, 14248–14250, DOI: 10.1103/PhysRevB.43.14248.

(S6) Neergaard Waltenburg, H.; Yates, J. Surface Chemistry of Silicon. *Chem. Rev.* **1995**, *95*, 1589–1673, DOI: 10.1021/cr00037a600.

(S7) Yokoyama, T.; Takayanagi, K. Anomalous flipping motions of buckled dimers on the Si(001) surface at 5 K. *Phys. Rev. B* **2000**, *61*, R5078–R5081, DOI: 10.1103/PhysRevB.61.R5078.

(S8) Hata, K.; Yoshida, S.; Shigekawa, H. $p(2 \times 2)$ Phase of Buckled Dimers of Si(100) Observed on n-Type Substrates below 40 K by Scanning Tunneling Microscopy. *Phys. Rev. Lett.* **2002**, *89*, 286104, DOI: 10.1103/PhysRevLett.89.286104.

(S9) Ramstad, A.; Brocks, G.; Kelly, P. J. Theoretical study of the Si(100) surface reconstruction. *Phys. Rev. B* **1995**, *51*, 14504–14523, DOI: 10.1103/PhysRevB.51.14504.

(S10) Guo, C.-S.; Hermann, K.; Zhao, Y. Dynamics and Energetics of Reconstruction at the Si(100) Surface. *J. Phys. Chem. C* **2014**, *118*, 25614–25619, DOI: 10.1021/jp509095t.

(S11) Brown, D. L.; Jones, K. S.; Phillpot, S. R. Lattice stability and point defect energetics of $TiSi_2$ and TiGe2 allotropes from first-principles calculations. *J. Appl. Phys.* **2021**, *129*, 085104, DOI: 10.1063/5.0029990.

(S12) Beyers, R.; Sinclair, R. Metastable phase formation in titanium-silicon thin films. *J. Appl. Phys.* **1985**, *57*, 5240–5245, DOI: 10.1063/1.335263.

(S13) Yan, Z. H.; Oehring, M.; Bormann, R. Metastable phase formation in mechanically alloyed and ball milled Ti–Si. *J. Appl. Phys.* **1992**, *72*, 2478–2487, DOI: 10.1063/1.351594.

(S14) Zhang, S.-L.; Östling, M. Metal Silicides in CMOS Technology: Past, Present, and Future Trends. *Crit. Rev. Solid State Mater. Sci.* **2003**, *28*, 1–129, DOI: 10.1080/10408430390802431.
